# The impact of crying, sleeping, and eating problems in infants on childhood behavioral outcomes: A meta-analysis

**DOI:** 10.3389/frcha.2022.1099406

**Published:** 2023-02-14

**Authors:** Britta Galling, Hannah Brauer, Pia Struck, Amanda Krogmann, Mirja Gross-Hemmi, Alexander Prehn-Kristensen, Susanne Mudra

**Affiliations:** ^1^ Department of Child and Adolescent Psychiatry, Psychosomatic Medicine and Psychotherapy, Charité-Universitätsmedizin Berlin, Berlin, Germany; ^2^ Institute of Child and Adolescent Psychiatry, Centre for Integrative Psychiatry, School of Medicine, University Medical Center Schleswig-Holstein – Campus Kiel, Kiel, Germany; ^3^ Department of Psychology, University of Hildesheim, Hildesheim, Germany; ^4^ University Hospital Hamburg-Eppendorf, Hamburg, Germany; ^5^ Swiss Paraplegic Research, Guido A. Zäch Institute, Nottwil, Switzerland; ^6^ Department of Psychology, Faculty of Human Sciences, MSH Medical School Hamburg - University of Applied Sciences and Medical University, Hamburg; ^7^ Department of Child and Adolescent Psychiatry, Psychotherapy and Psychosomatics, University Medical Center Hamburg-Eppendorf, Hamburg, Germany

**Keywords:** regulatory problems, early infancy, excessive crying, sleeping problems, eating problems, child behavior, ADHD

## Abstract

**Background:**

There is increasing evidence that regulatory problems (RPs), such as excessive crying, sleeping or feeding problems in infancy, could be associated with the development of behavioral problems in childhood. In this meta-analysis we aimed to investigate the strength and characteristics of this association.

**Methods:**

A systematic literature search (PubMed/PsycInfo, until 15/08/2021) for longitudinal prospective studies of infants with RPs and at least one follow-up assessment reporting incidence and/or severity of behavioral problems was conducted. The primary outcomes were (i) the cumulative incidence of behavioral problems in children (2–14 years) with previous RPs and (ii) the difference between children with/without previous RPs with regard to the incidence and severity of externalizing, internalizing and/or attention-deficit/hyperactivity disorder (ADHD) symptoms. Additionally, we analyzed behavioral problems of children with previous single, multiple or no RPs and with respect to age at follow-up. Subgroup and meta-regression analyses were added.

**Results:**

30 meta-analyzed studies reported on 34,582 participants (n_RP _= 5091, n_control _= 29,491; age: baseline = 6.5 ± 4.5 months, follow-up = 5.5 ± 2.8 years) with excessive crying (studies = 13, *n* = 1577), sleeping problems (studies = 9, *n* = 2014), eating problems (studies = 3, *n* = 105), any single (studies = 2, *n* = 201) or multiple RPs (studies = 9, *n* = 1194). The cumulative incidence for behavioral problems during childhood was 23.3% in children with RPs. Behavioral problems were significantly more pronounced in infants with RPs compared to healthy controls (SMD = 0.381, 95% CI = 0.296–0.466, *p* < .001), particularly with multiple RPs (SMD = 0.291, *p *= 0.018).

**Conclusions:**

Findings suggest that RPs in infancy are associated with overall behavioral problems (externalizing or internalizing behavior and ADHD symptoms) in childhood. Our data cannot explain linked developmental trajectories and underlying factors. However, detection of affected infants may help to adapt supportive measures to the individual familial needs to promote the parent-child-relationship and prevent the development of child behavioral problems from early on.

## Introduction

During the first years of life, the ability to self-regulate is one of the most important developmental tasks as it is closely related to the infant's general adaptability to its environment and ultimately its survival (1). Self-regulation entails the infant’s ability to control behavior, including physiological, sensory, motoric, attentional, and emotional processes, such as self-soothing, ingesting food, developing a sleep-wake regulation as well as attaining an alert state that enables social interaction (2). Besides maturation processes, the development of self-regulation is enabled by the primary caregivers, mostly the parents, embedded in a dyadic interaction. This reciprocal relationship includes infant self and parent-infant co-regulatory processes (3, 4). Thus, an infants's regulatory capacities can be seen as fundamental aspects of childhood development (5). However, some infants display dysregulation in these processes which are defined as difficulties in adjusting to the environment, the regulation of behavior, arousal, and self-soothing. These are labelled as regulatory problems (RPs), which are excessive crying, sleeping, or feeding difficulties (6).

According to the diagnostic classification of mental health and developmental disorders of infancy and early childhood (DC: 0–5), infants are diagnosed with primary “sleep, eating and crying disorders”, if the functioning of the infant, parent, or both is persistently impaired, and other diagnoses such as a sensory processing disorder are ruled out (7). RPs can either manifest themselves as a single problem (e.g., excessive crying only) or co-occur as multiple RPs (8, 9). Numerous studies have shown that an infant's capacity to regulate their own behavior in terms of crying, eating, and sleeping problems are strong predictors of developmental, cognitive, behavioral, and emotional difficulties throughout childhood, including aggression, attention problems, anxiety, or depression (10–13). While most RPs are temporary and disappear during infant development, some RPs can persist or even exacerbate and lead to long-term consequences (13–15).

There is increasing evidence that RPs such as excessive crying, sleeping, or feeding problems in infancy could be associated with the development of behavioral problems in childhood. A previous meta-analysis by Hemmi et al. (16) on this association found small effect sizes for internalizing behavior and ADHD and medium effect sizes for externalizing behavior.

Primary study data suggest that single RPs such as excessive crying (17), eating (18) or sleeping problems (19) are associated with a higher risk of behavioral problems in infancy. Combinations of RPs show similar relationships: Persistent crying problems co-occurring with sleeping or eating problems at 3–6 months were found to be associated with externalizing problems at age 8 to 10 (13), and multiple RPs at 6 months were found to be associated with internalizing, externalizing, and general behavior problems at age 5 and 14 (20). Moreover, one study found crying and sleeping difficulties to be linked with disorganized attachment (21). Research also found infants with RPs to be at an increased risk of developing deficits in social skills (9) and cognitive development (22) at preschool age.

Risk factors for RPs include parent-related factors, such as emotional or professional distress (23–26) or lack of parental intuitive skills (7). On the other hand, pregnancy (27–29), birth (14, 26, 30) and more infant-related factors (6, 23) also play a crucial role. Consequences of RPs include high level of distress for the family and are associated with psychosocial problems such as family disruption, parental insecurity, depression and anxiety, or lack of self-efficacy (31, 32). These can result in parents seeking professional help more frequently and considerably higher health care costs (33, 34), but may also lead to emotional and/or physical maltreatment of infants (35), such as the shaken baby syndrome (36).

A better understanding of the association of infant RPs and the risk of behavioral problems during childhood is of great importance to improve early detection and intervention. This is particularly true, since lasting behavioral problems during childhood have been linked to a higher risk for impairments in academic achievement (37) and subsequent mental illness (38).

Since several longitudinal studies have been published since 2,011 and provided additional evidence, we aimed to update and complement the findings of a previous meta-analysis (16). Particularly, we added the cumulative incidence as primary outcome and adjusted some analyses in view of methodological considerations. For example, we aimed to ensure a more distinct classification of outcomes by merging available data as accurately as possible. That is, we chose one outcome per study with *à priori* specified outcome definitions in contrast to the previous meta-analysis that combined ≥ one study outcome in the same meta-analysis. Moreover, we added several subgroup analyses and metaregression analyses. We compared for example behavioral outcomes of those with multiple vs. single RPs to find out whether behavioral problems are more likely in children with previous multiple RPs compared to those with single RPs. Also we analyzed the effect of the follow-up age to look for vulnerable time points for the development of behavioral problems (for details see methods).

Overall, our analysis aimed to allow for a better understanding of the strength and the characteristics of the association of RPs in infancy with the development of behavioral problems in childhood.

## Methods

This systematic review was conducted in accordance with the Preferred Reporting Items for Systematic Reviews and Meta-Analyses (PRISMA) standard (39, 40).

### Literature search

Two independent authors (PS, HB) searched PubMed/MEDLINE and PsycInfo through 15/08/2021 without language restrictions, supplemented by a manual review of reference lists from eligible publications and relevant reviews and meta-analyses. The search terms used were: (“infant crying” OR “crying problem*” OR “excessive crying” OR “persistent crying” OR “feeding problem*” OR “feeding disorder” OR “refusal to eat” OR “picky” OR “choosy” OR “infant sleep” OR “sleeping disorder*” OR “regulation disorder*” OR “regulatory problem*” OR “regulatory disorder*”) and (“attention-deficit” OR “attention deficit” OR hyperactivity OR ADHD OR ADD OR hyperkinetic OR “behavioral problems” OR “behavioral outcome” OR “emotional problem” OR “internalizing” OR “externalizing” OR “dysregulated behavior” OR dysregulation OR anxiety OR fear OR psychopathology OR “clinical symptoms” OR preschool OR “growing up” OR follow-up OR “follow up” OR longitudinal OR prospective OR “mental health” OR “epidemiology”). Authors were contacted for additional information.

### Inclusion criteria

Inclusion criteria were: (i) prospective study, (ii) reporting on **≥**20 children with (iii) RPs regarding crying, sleeping, and/or eating problems (study-defined, see Table 1) (iv) during infancy (≤18 months of age (41)), and (v) more than one follow-up assessment during childhood (2–14 years of age) (vi) reporting on behavioral problems such as externalizing, internalizing, and/or ADHD symptoms. We did not include studies that only reported RPs as outcome at follow-up.

**Table 1:** Study and sample characteristics.

Study	Study quality (NOS)	N (baseline)		Age (mean)		Sample		Analyzed follow-up outcomes
		RPs	HC	Baseline (months)	Follow-up (years)	Clinical vs. community	RP assessment criteria	Outcomes (Categorization)
**Crying**								
Bell 2018 (Australia)	7	99	182	1.7	2	Clinical	Wessel criteria	CBCL (EXT, INT)
Canivet 2000 (United States)	7	52	102	3	4	Community	Wessel criteria	RCBQ (EXT, INT)
DeSantis 2004 (United States)	4	165	–	2	5.6	Clinical	N hours crying/fussing	CBCL (overall BP, EXT, INT, ADHD)
Elliott 1997 (Canada)	6	10	72	1.8	3	Community	Wessel criteria	CBCL (overall BP)
Neu 2003 (United States)	5	20	20	2.5	7	Clinical	Crying for ≥2.8 h/d, ≥ 3 days	CBCL, DICA-R (EXT, INT, ADHD)
Papousek 2001 (Germany)	6	83	57	4.1	2.5	Clinical	Wessel criteria	CBCL (EXT, INT)
Rao 2004 (Norway)	7	63	264	9	5	Community	Daily uncontrolled crying, ≥ 2 weeks	PIC (ADHD)
Rautava 1995 (Finland)	6	338	866	3	3	Community	Questionnaire-based colic d/o	Parent-Report (EXT)
Santos 2015 (Brazil)	7	437	3237	3	4	Community	> avg. crying at same age	CBCL (overall BP, EXT, INT)
Savino 2005 (Italy)	7	52	51	2	10	Clinical	Crying avg. 4 h/d, > 4 d/week	Clinical evaluation (EXT)
Smarius 2017 (Netherlands)	7	102	3287	3	5	Community	Crying avg. ≥3 h/d/week	SDQ (overall BP, EXT, INT)
Wake 2006 (Australia)*	6	55	313	3	2	Community	Sleep and cry-fuss problems (Parent-Report)	CBCL (overall BP, EXT, INT)
Wolke 2002 (Germany)	5	101	64	3.8	9.7	Clinical	Modified Wessel criteria	SDQ (overall BP, EXT, INT, ADHD)
**Sleeping**								
Cook 2019 (Australia)*	7	446	647	12	5; 11	Community	Questionnaire-based sleep problem	SDQ (overall BP)
Cook 2020 (Australia)	9	283	360	7	4; 10	Community	Awakenings ≥3 times/night in the last week	DAWBA (ADHD, EXT, INT)
O’Callaghan 2010 (Australia)	7	754	2943	6	5; 14	Community	Sleeplessness most days/a few times a week	CBCL (ADHD)
Price 2012 (Australia)	4	225	–	7	6	Community	Parent-reported sleep problem	SDQ (overall BP, EXT, INT)
Scher 2005 (Israel)	6	13	12	12	3.5	Community	Night waking and settling difficulties	CBCL (overall BP)
Thunström 2002 (Sweden)	7	27	25	8.5	5.5	Community	≥15 min. to fall asleep; awakenings ≥3 times/night ≥5 nights/week. for ≥6 months	Standardized Parental Interview, Standardized Clinical Interview (EXT, ADHD)
Zuckerman 1987 (United Kingdom)	6	56	–	8	3	Community	≥1 h to settle after waking; awakenings ≥3 times/night; problem causing severe disruption to the mother's sleep	BSQ (EXT, INT, ADHD)
Östberg 2011 (Sweden)*	3	125	227	13	7.3	Clinical	Referred (sleep problem)	Connor's scale, RCBQ (EXT, INT)
Wake 2006 (Australia)*	6	85	313	8	2	Community	Sleep and cry-fuss problems (Parent-Report)	CBCL (overall BP, EXT, INT)
**Eating**								
Dahl 1992/1994 (Sweden)	6	25	240	7.8	4; 9.6	Clinical	Refusal to eat and/or eating problem ≥1 month	Rutter PBQ, HSQ (overall BP, ADHD)
Motion 2001 (United Kingdom)	5	28	10669	6	3.9	Community	Eating difficulties for 4 weeks	SDQ (ADHD, EXT)
Östberg 2011 (Sweden)*	3	52	227	13	7.6	Clinical	Referred (eating problem)	Connor's scale, RCBQ (EXT, INT)
**Crying/Sleeping/Eating**								
Becker 2004 (Germany)	9	175	264	3	2; 4.5; 8; 11	Community	>1 SD ≥ mean for one factor=SRP, >1 SD ≥ mean for irritable and somatic functioning = MRP	MPI (overall BP, ADHD)
Bilgin 2020 (Germany)	7	469	977	5	8	Community	Cry duration ≥2 h/d, cry amount > avg., difficult to soothe; wakes up ≥2 times/night, ≥15 min at night; eating difficulties, vomiting, disordered mouth/tongue movement	CBCL (ADHD)
Cook 2019 (Australia)*	7	59	647	12	5; 11	Community	Presence and severity of sleep problems, excessive crying; coughed/choked food; global temperament, mood swing	SDQ (overall BP)
DeGangi 1993 (United States)	6	9	13	9.5	4	Clinical	>20 min. to fall asleep, >2 waking/night	SHQ (overall BP, ADHD)
DeGangi 1996 (United States)	5	13	-	18.5	3	Clinical	>20 min. to fall asleep, >2 waking/night	CBCL, expert observation (overall BP, ADHD)
DeGangi 2000 (United States)	5	22	38	18.5	3	Clinical	>20 min. to fall asleep, >2 waking/night	SHQ, clinical diagnosis (overall BP)
Forsyth 1991 (United States)	7	115	205	4	3.5	Community	parent-reported sleep problem	CBCL (overall BP)
Hyde 2012 (Australia)	7	480	4356	6	5; 14	Community	Colic, sleeplessness, eating problems, overactivity	CBCL (overall BP, EXT, INT)
Östberg 2011 (Sweden)*	3	53	227	13	7.5	Clinical	Referred (sleep/eating problem)	Connor's sale, RCBQ (EXT, INT)
**All studies**								
Total	6	5091	29,491	6.5***	5.5***			

*Due to different RPs group comparisons, these studies are listed multiple times, however they were only considered once for the calculation of total means and Ns (“All studies”).

**Of the RPs group.

***Not adjusted for sample size (adjusted means baseline: 5.4months; follow-up: 5.8years). ADHD = Attention-deficit-hyperactivity disorder; avg. = average; BP = behavioural problem; BSQ = Behavioral Style Questionnaire; CBCL = Child Behavior Checklist; DAWBA = Development and Well-Being Assessment; DICA-R = Diagnostic Interview for Children and Adolescents-Revised; EXT = externalizing problems; HSQ = Home and School Questionnaire; INT = internalizing problems; MPI = Mannheim Parent Interview; MRP = multiple regulatory problems; N = number of subjects; NOS = Newcastle-Ottawa-Scale; PIC = Personality Inventory for Children; Rutter PBQ = Rutter Preschool Behavior Questionnaire; RCBQ = The Rutters Children's Behaviour Questionnaire; RPs = regulatory problems; SDQ = Strengths and Difficulties Questionnaire; SHQ = Sensorimotor History Questionnaire; SRP = single regulatory problem.

If studies with a community sample only reported continuous measures of RPs of the complete sample at baseline and did not distinguish between RPs and healthy controls (HCs), they were not included. We also excluded studies restricted to children with any kind of disability, pervasive developmental or autism spectrum disorder or those investigating other clinical outcomes only (e.g., eating disorder, obesity, developmental disorder, neurological outcomes). If studies reported on HCs, these data were used irrespective of whether they were assessed prospectively or retrospectively.

### Outcomes and outcome measures

Primary outcomes were (i) the cumulative incidence of overall behavioral problems of infants with any RPs (single and multiple) during infancy and (ii) the overall behavioral problems of infants with any RPs (single and multiple) compared to HCs. The outcome of overall behavioral problems summarized the amount and severity of symptoms for the primary and secondary outcomes using the total behavioral problem scores (such as CBCL total score). If those measures were not available, measures of externalizing (preferred), internalizing, and/or ADHD symptoms were supplemented. Both continuous and categorical outcomes were included (42, 43).

Secondary outcomes included (iii) overall behavioral problems of infants with single RPs compared to HCs, (iv) overall behavioral problems of infants with multiple RPs compared to healthy controls, and (v) overall behavioral problems of infants with multiple RPs compared to those with single RPs.

Other outcomes included the cumulative incidence of overall behavioral problems in infants with any RPs and HCs as well as overall behavioral problems and in-between group differences (as described above) of (vi) externalizing problems, (vii) internalizing problems, and (viii) ADHD symptoms separately. If a study reported multiple measures for one outcome, we chose the scale used most often in the overall study sample to ensure homogeneity (for details see Table 1 and Online Resource 1 Supplementary Table S1).

### Data extraction

Data of each study were independently identified and extracted by more than 2 authors (HB; PS); inconsistencies were resolved involving a third reviewer (BG). Unadjusted outcome data were preferred. When more than 2 samples with different symptom severity of the same RPs were studied, we extracted data from the sample with the most severely reported symptoms. If continuous and categorical data were reported, continuous data were preferred. Whenever data were missing, authors were contacted for more information.

In the case of overlapping samples, we included the most suitable data (largest sample size and/or matching inclusion criteria). In this context, we decided in one case (18) to extract outcomes from a limited study sample that better matched the predefined follow-up age (9.5 years instead of 4 years). All outcomes were extracted separately for the following groups: (a) any RPs, (b) single RPs, (c) multiple RPs, and (d) HCs. In the group of any RPs, outcomes of infants with single or multiple RPs were pooled, and the incidences/severity scores were merged.

If multiple follow-up assessments were available, we preferred those at age 5–11 years for the main analysis, as most children were assessed at that age. In case of multiple follow-up assessments between age 5–11 years, the later time point was preferred. Additionally, all other follow-up time points were extracted for a separate analysis of age groups. For the studies with multiple follow-up time points, we extracted and used multiple data from different time points. For the category “definition of RP” a studies definition was considered strict, if the assignment to the RP group was based on a structured interview or questionnaire, and it was considered lenient if it was based on a one-item parent report.

### Assessment of study quality

Study quality was evaluated *via* the Newcastle-Ottawa Scale (NOS), which is a scale used for assessing the quality of nonrandomized studies in meta-analyses in the three categories 1) selection of study groups, 2) comparability of the groups and 3) ascertainment of outcome of interest. Data regarding the study quality of each study were independently identified and extracted by ≥2 authors (HB; PS). The overall result is indicated by the NOS score, where a score of ≥7 out of 9 indicates high study quality (44).

### Data analysis

The cumulative incidence was computed as the number of children with mental or behavioral problems at follow-up divided by the total number of individuals in the population at risk.

Between-group differences were described for each outcome where more than 2 studies were available using the standardized mean difference (SMD). The SMD was either extracted directly or calculated from means, standard deviations (SDs) and sample sizes, odds ratios, F-statistics, or correlation coefficients (45). The SMD was adjusted using the small sample size bias correction (Hedges' g) (42). SMDs were considered small if between 0.2–0.49, medium if between 0.50–0.79, and large if ≥0.80 (46). Data were analyzed using R (R Core Team, 2019) and Comprehensive Meta-Analysis Version 3 (Borenstein, 2013). All analyses used a random effects model (47), were two-sided, with alpha = 0.05, and were presented as point estimates and corresponding 95% confidence intervals (CIs). In addition, *p*-values were used to describe the test-for-null outcome effects. Heterogeneity among studies was assessed with *I*^2^ value (48), with *p* < .05 and *I*^2 ^≥ 50% indicating significant heterogeneity. Publication bias was assessed for the primary outcomes by using funnel plots and Egger's regression test for funnel plot asymmetry (42, 49) for analyses with ≥10 studies. In addition, we used the trim and fill method which yields an estimate of the effect size after the publication bias (50) and the fail-safe test (estimated number of studies needed in order to obtain a non-significant result).

The following subgroup analyses were added for all outcomes: (i) sample (community vs. clinical), (ii) RP definition (strict vs. lenient), (iii) type of RPs (crying vs. sleeping vs. eating; only for comparison of single RPs vs. multiple and single RPs vs. HC), and (iv) age at baseline (≤6 vs. > 6 months). We conducted random effects meta-regression analyses to identify potential moderators including (i) age at baseline, (ii) age at follow-up, (iii) percentage male, (iv) sample size, and (v) study quality (NOS). To account for a potential change of symptoms with age, we added an additional analysis for any RPs looking at the overall behavioral problems reported for the following age groups only: 2–6, 7–10, 11–14.

## Results

### Search results

**Figure 1:**
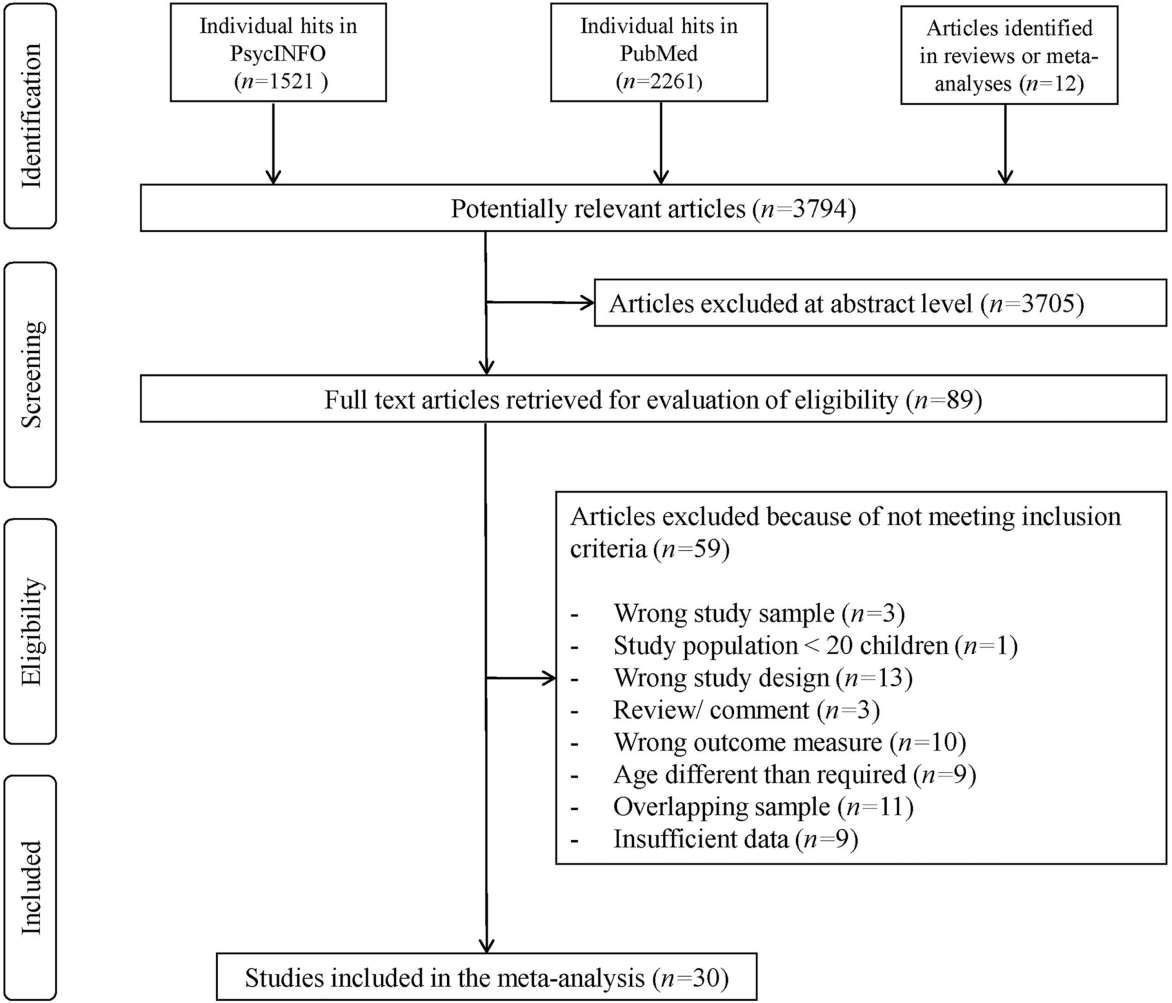
PRISMA diagram of the systematic literature search.

The initial search resulted in 3,794 hits. Altogether, 3,705 studies were excluded on the title/abstract level. Of the remaining 89 references 59 articles were excluded after full text review, yielding 30 studies (10, 12, 13, 15, 17–20, 32, 51–69) (Figure 1) that were included in this meta-analysis.

### Study characteristics

A total of 30 studies reported on 34,582 participants (RPs: *n* = 5091, control: *n* = 29,491; baseline = 6.5 ± 4.5 months, follow-up = 5.5 ± 2.8 years, male = 52%). Single RPs were examined in 25 studies (*n* = 3897; crying problems: studies = 13, *n* = 1577; sleeping problems: studies = 9, *n* = 2014; eating problems: studies = 3, *n* = 105; not specified: studies = 2, n = 201), multiple RPs in 9 studies (*n* = 1194). Five studies reported co-occurrence of RPs but only analyzed the outcome of single RPs (11, 13, 56, 60, 62) (Table 1, Online Resource Supplementary Table S1).

The overall study quality was high with a mean NOS score of 6.2 ± 1.3 (median = 6, 95% CI = 6–7) and a NOS ≥7 (indicating high study quality) in 13 of 30 studies (43%) (Online Resource Supplementary Table S2).

### The cumulative incidence of overall behavioral problems in children with previous RPs and HCs

The meta-analytically calculated cumulative incidence of overall behavioral problems in children with previous RP was 0.233 (95% CI = 0.179–0.298, studies = 18, *n* = 2873), indicating that 23.3% of those with RPs during infancy developed behavioral problems later (Table 2). Of note, is that this is nearly 4 times more frequent than in HCs that had a cumulative incidence of overall behavioral problems of 0.067 (95% CI = 0.043–0.104, studies = 10, *n* = 3699). No significant subgroup differences or moderating effects emerged in children with previous RPs, except for smaller sample sizes that were associated with higher overall behavioral problems (*p* = 0.022) (Online Resource Supplementary Table S3).

**Table 2:** Primary outcomes and specific behavioral problem outcome categories.

OVERALL BEHAVIORAL PROBLEMS							
CUMULATIVE INCIDENCE				COMPARISON OF ANY RPs TO HEALTHY CONTROLS			
SUB-GROUP	N (*n*)	Incidence	N (*n*)	SMD	Result: *p*-value	Heterogeneity	
						*p*-value	*I* ^2^
All Studies	18 (2873)	0.233	26 (31,177)	**0**.**381**	**<0**.**001**	<0.001	56.9
Community sample	11 (2603)	0.201	19 (30,057)	**0**.**348**	**<0**.**001**	0.070	34
Clinical sample	7 (270)	0.300	7 (1120)	**0**.**685**	**<0**.**001**	<0.001	78.2
Strict^a^	16 (1682)	0.234	24 (23,806)	**0**.**381**	**<0**.**001**	<0.01	65.4
Lenient^b^	2 (1191)	0.233	2 (7371)	**0**.**333**	**<0**.**001**	0.84	0.00
Crying problems	6 (609)	0.268	11 (9116)	**0**.**493**	**< 0.001**	<0.001	66.6
Sleeping problems	4 (1060)	0.231	7 (5319)	**0**.**266**	**< 0.001**	0.206	29.1
Eating problems	0		3 (11,228)	0.231	0.072	0.256	26.6
Single RPs^c^	13 (2182)	0.228	23 (26,789)	**0**.**377**	**<0**.**001**	56.1	<0.001
Multiple RPs^c^	6 (687)	0.248	8 (7474)	**0**.**415**	**<0**.**001**	77.1	<0.001
≤6 months	8 (892)	0.274	13 (11,012)	**0**.**405**	**<0**.**001**	<0.001	63.7
>6 months	10 (1981)	0.208	13 (21,585)	**0**.**355**	**<0**.**001**	0.020	50.1
**EXTERNALIZING PROBLEMS**							
All Studies	11 (1544)	0.201	16 (25,702)	**0**.**362**	**<0**.**001**	0.001	62.065
**INTERNALIZING PROBLEMS**							
All Studies	8 (1443)	0.160	12 (13,865)	**0**.**343**	**<0**.**001**	0.602	0.000
**ADHD**							
All Studies	8 (1065)	0.242	11 (15,019)	**0**.**461**	**<0**.**001**	0.071	41.701

SMDs (standardized mean differences) >0 indicate that a specific continuous outcome (e.g., symptom severity) was more pronounced in those with regulatory problems (RPs). *P*-values ≤ 0.05 were considered statistically significant and are marked in bold together with the respective SMD. For more details about overall behavioral problems see Online Resource 1 Supplementary Table S3 and Online Resource Supplementary Table S5 for subgroup analyses of the outcomes externalizing problems, internalizing problems and ADHD; CI = confidence interval; Coeff = coefficient; N = number of comparisons; n = number of subjects; n/a = not applicable; SMD = standardized mean difference.

^a^ Strict definition = structured interview or questionnaire based.

^b^ Lenient definition = one-item parent report.

^c^ Including separate single and multiple RPs within a study.

### Overall behavioral problems of infants with RPs compared to HCs

**Figure 2:**
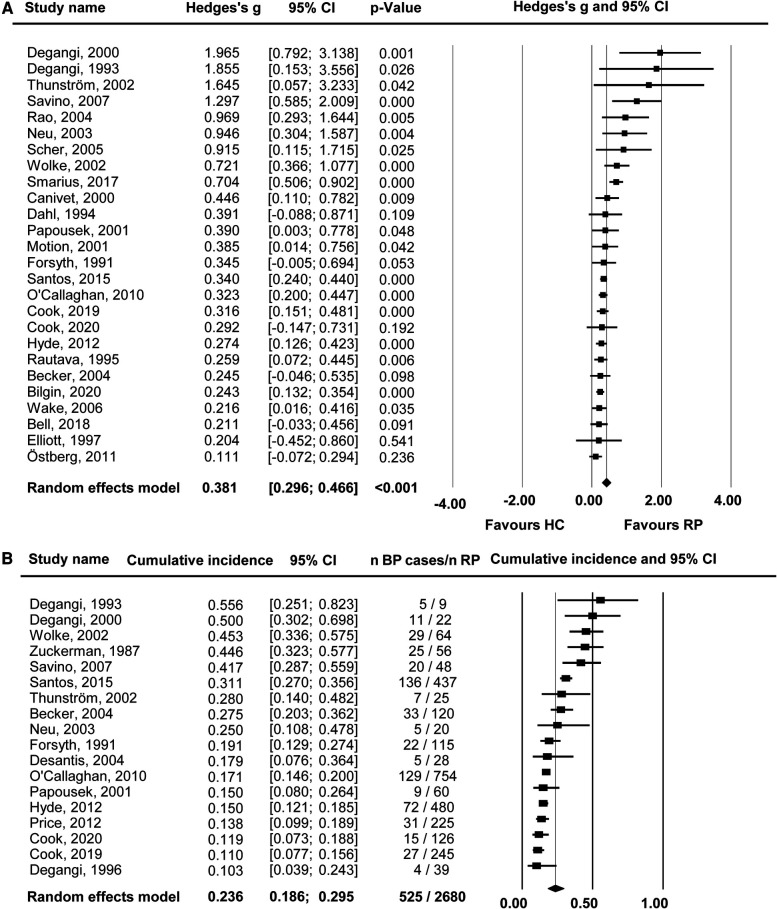
Overall behavioural outcomes (any regulatory problems vs. healthy controls). Forest plot of (**A**) standardized mean difference (Hedges's g) and (**B**) cumulative incidence for overall behavioural problems in children with any regulatory problems (RPs) in infancy vs. healthy controls (HC). SMDs > 0 indicate that a specific outcome was more pronounced in the RPs than the HC group. Black whiskers mark the 95% confidence interval (CI).

When meta-analytically comparing the overall behavioral problems of children with previous single and multiple RPs during infancy to HCs (studies = 26, *n* = 31,177), those with RPs had significantly more behavioral problems (SMD = 0.381, 95% CI = 0.296–0.466, *p* ≤ 0.0001) (Figure 2).

The Egger's test (intercept = 1.422, 95% CI = 0.6–2.25, *p *= 0.003) indicated potential publication bias. After adjustment for 6 potentially missing studies using the trim-and-fill method, the SMD decreased to 0.339 (95% CI = 0.243–0.434).

No significant effects emerged in the subgroup or meta-regression analyses. However, effects sizes were particularly high (SMD = 0.685, 95% CI = 0.295–1.074, *p* ≤ 0.001) in the clinical sample compared to the community sample (SMD = 0.348, 95% CI = 0.275–0.422, *p* ≤ 0.001) (Table 2). Effect sizes were alike, regardless of the follow-up age (see Table 3).

**Table 3:** Comparison of overall behavioral problems in single RPs vs. multiple RPs vs. healthy controls and different age groups at follow-up.

OVERALL BEHAVIORAL PROBLEMS SINGLE RPs vs. MULTIPLE RPs vs. HEALTHY CONTROLS					
COMPARISON	N (*n*)	SMD	Result: *p*-value	Heterogeneity	
				*p*-value	*I* ^2^
Single RPs vs. HCs	23 (26,789)	**0**.**372**	**<0.001**	<0.01	56.1
Multiple RPs vs. HCs	8 (7474)	**0**.**419**	**<0.001**	<0.001	77.1
Single RPs vs. Multiple RPs	4 (961)	0.149	0.463	<0.001	82.5
**OVERALL BEHAVIORAL PROBLEMS OF ANY RPs vs. HEALTHY CONTROLS AND DIFFERENT AGE GROUPS AT FOLLOW-UP**					
3–6 years	21 (31,495)	**0**.**363**	**<0.001**	0.008	48.1
7–10 years	7 (4138)	**0**.**334**	**<0.001**	0.201	35.2
11–14 years	4 (9347)	**0**.**395**	**<0.001**	0.002	48.9

SMDs (standardized mean differences) > 0 indicate that a specific continuous outcome (e.g., symptom severity) was more pronounced in those with regulatory problems (RPs). *P*-values ≤ 0.05 were considered statistically significant and are marked in bold together with the respective SMD; CI = confidence interval; Coeff = coefficient; HCs = healthy controls; N = number of comparisons; n = number of subjects; SMD = standardized mean difference.

### Comparison of single and multiple RPs

The overall behavioral problems of children with single RPs (studies = 23, *n* = 26,789) and with multiple RPs during infancy (studies = 8, *n* = 7474) were significantly more pronounced compared to HCs (single RPs: SMD = 0.372, 95%, CI 0.281–0.462, *p* ≤ 0.0001; multiple RPs: SMD = 0.419, 95% CI 0.200–0.639, *p* ≤ 0.001). Children with multiple RPs during infancy did not show significantly more overall behavioral problems than those with single RPs (studies = 4, *n* = 961, SMD = 0.149, 95% CI = −0.250–0.549, *p* = 0.463) (see Online Resource Supplementary Table S4).

### Type of rp: crying vs. Sleeping vs. Eating problems

The overall behavioral problems of children with excessive crying (SMD = 0.493, 95% CI = 0.336–0.651, *p* ≤ 0.001) and sleeping problems (SMD = 0.266, 95% CI = 0.138–0.395, *p* ≤* *0.001) were significantly more pronounced compared to HCs, while no significant effect on childhood outcomes emerged in those with eating problems during infancy (SMD = 0.231, 95% CI = −0.021–0.482) (see Table 2 and Online Resource Supplementary Table S3). In-between subgroup differences (crying vs. sleeping vs. eating problems) were not significant (*p* = 0.128).

### Externalizing problems, internalizing problems, and ADHD

Children with previous RPs were also more frequently affected by externalizing problems (RPs: cumulative incidence = 0.201, 95% CI = 0.141–0.279; HCs: cumulative incidence = 0.067, 95% CI = 0.028–0.151, SMD = 0.362, 95% CI = 0.253–0.472, *p *= 0.001), internalizing problems (RPs: cumulative incidence = 0.160, 95% CI = 0.120–0.209; HCs: cumulative incidence = 0.083, 95% CI = 0.063–0.109, SMD = 0.343, 95% CI = 0.284–0.403, *p *≤ 0.001), and ADHD (RPs: cumulative incidence = 0.242, 95% CI = 0.157–0.354; HCs: cumulative incidence = 0.076, 95% CI = 0.026–0.201, SMD = 0.461, 95% CI 0.317–0.605, *p *= 0.071) (Table 2). For subgroup and meta-regression analyses see Online Resource Supplementary Table S5.

## Discussion

This meta-analysis aimed to comprehensively quantify the association between RPs in infancy and the occurrence of behavioral problems during childhood. A total of 30 prospective longitudinal studies were included to examine the association between RPs and behavioral problems across a wide range of clinically relevant outcomes, including overall problem behavior, externalizing behavior, internalizing behavior, and ADHD symptoms.

Results indicate a cumulative risk of 23.3% for children with RPs compared to 6.7% for HCs to develop overall behavioral problems throughout their childhood (2–14 years). Considering that the incidence of behavioral problems is nearly 4 times more frequent after RP's (even though the effect sizes were small to medium only), early prevention could have a substantial effect by shifting the distribution in the total population for millions of children worldwide.

Our analysis found that behavioral problems after RPs were extensive and include externalizing behavior, internalizing behavior, and ADHD which is in line with the results of the meta-analysis by Hemmi et al. (16). We also found comparable effect sizes regardless of the follow-up age, indicating that the behavioral problems that are reported in young children who had already suffered from RPs, do not seem to improve with age.

The underlying reasons that might explain the association between RPs and behavioral problems during childhood cannot be explained by our analysis. From a relational perspective, due to the complex early interplay between parents and infants in the development of self-regulation, one can differentiate behavioral, environmental, developmental, interactional, and mental health variables on the parental and infant side which might contribute to ongoing behavioral problems later on (4, 7, 70).

Our results are in line with a cascade model of child development: Early problems with regulation may provide the starting point of a trajectory of dysregulated behaviors, such as problems to sustain attention (51, 71). Consequently, RPs that develop at an early stage in life may affect learning processes and the ability to regulate emotions and behaviors later in childhood as well, predicting a higher risk for clinical disorders in childhood and adolescence (72). Developmental milestones might not be accomplished, leading to continuing deficits reflected in later behavioral problems (13). In contrast to this model, which suggests that a high amount of problems during infancy may lead to more severe problems during childhood, our results do not indicate a significant difference with regards to behavioral symptom severity during childhood depending on the extent of RP (single or multiple RPs) during infancy.

However, these results should be interpreted carefully as this analysis was based on four studies only: Three out of these four studies (11, 53) found evidence that multiple RPs were associated with more behavioral problems than single RPs, while data of one study presented opposite results (73), leading to an overall non-significance in this meta-analytic comparison. Looking at single RPs in sub-group analyses for overall behavioral problems, SMDs were 0.493 for crying problems and 0.266 for sleeping problems. It might be that the overall severity of single and/or multiple RPs – other than the type and amount of RPs – other than the type and amount of RPs – might be of importance: The association of RPs and behavioral problems was way more pronounced in the clinical sample (SMD = 0.685) compared to the community sample (SMD = 0.348). These results are underlined by a study that found “persistent excessive crying” to be associated with a higher risk to develop multiple RPs (74).

From a neurobiological perspective, RPs in infancy, attention problems, and internalizing behavior in childhood have been associated with dysregulation of the hypothalamic-pituitary-adrenal (HPA) axis (67, 75, 76). Furthermore, more deficient self-regulation has been demonstrated in a particular gene polymorphism of the dopaminergic system contributing to multiple RPs (77), ADHD, and externalizing behavior in childhood (78). Thus, self-regulatory problems primarily obvious as RPs in infancy may be expressed as other forms of emotional dysregulation in early childhood, such as disrupted mood and anger disorder, anxiety, impulsivity or hyperactivity in preschoolers, with an elevated risk for behavioral problems in childhood (7, 73, 79).

Evidence suggests preterm birth, infant temperament or general cognitive impairment as precursors of behavioral or attention problems which have been also associated with an elevated risk for RPs (73, 80–83). These results are affirmed by our subgroup analysis for overall behavioral problems that showed stronger effect sizes in clinical samples compared to community samples. A possible explanation could be that clinically referred children might already have been exposed to multiple risk factors, such as obstetric adversities or severe relational or psychosocial family problems (16).

However, not all infants with early vulnerability and RPs develop behavioral problems in childhood. To investigate associated trajectories or underlying factors more precisely, longitudinal studies are needed which take the mutual parent-child-model with different factors from both perspectives into account and include the prenatal period, which might shape infant´s regulatory skills before birth (4, 27, 84). Besides the challenges that cause distress in parents of an infant with primary self-regulatory deficits, there are several parental factors that may promote RPs, affect the parent-infant interaction, and need further attention in future studies. Among them, parental mental health, particularly maternal depression, hostility and anxiety, parental mentalization, and the quality of parenting behavior are central (4, 7, 23, 70). If parents are less able to co-regulate and compensate an infant´s difficultness, or cannot read the infant's signals and react in a prompt and sensitive way to it due to their own impairment, there might be an elevated risk for persistent RPs and later behavioral or attentional problems (4, 70, 84, 85).

### Strengths and limitations

The findings of the conducted meta-analysis are consistent with the existing meta-analysis by Hemmi et al. (16) and extend the scope of the negative impact of RPs on childhood behavioral problems. A considerable effort was made to include as much data as possible in this meta-analysis whilst maintaining strict inclusion criteria: Additional information from eleven studies was included in this meta-analysis. Using strict inclusion criteria and methodological rigor, we aimed to rule out as many sources of potential bias as possible. In this context, we excluded three studies that Hemmi et al. (16) had included (including a control group with transient RPs and a follow-up age younger than two years). Moreover, we expanded methods by also reporting on the cumulative incidence of behavioral problems during childhood.

There was high heterogeneity in the data. Identified studies were heterogeneous with respect to sample characteristics, RP definition, measurement instruments, number of subscales, outcomes, and follow-up duration (range 2.5–11 years) which likely contribute to the heterogeneity of the results. Many different forms of defining and assessing RPs existed across countries and centers. Consequently, the use of standardized tools that focus on parent and infant behaviors for enhanced comparability in further research is needed. Since most studies assessed infant RPs and child behavior using parent reports rather than objective measures or clinician observation, a reporting bias might influence the results. For example, maternal “overrating” of the children's behavior might be rooted in maternal stress and/or the continued perception of the child as being difficult (20, 73). Therefore, in future research more objective and multi-informant measures of child behavior (e.g., clinical observation, teacher reports), parental characteristics, and the parent-infant interaction should be used (86).

Moreover, some studies showed co-occurrence of RPs but only reported behavioral problem outcomes for single RPs and did not control for any other RPs. Previous research found that crying, sleeping, and eating problems often coexist (6, 13). Hence, non-reported or non-assessed co-occurrence of RPs might lead to a biased conclusion regarding the effects of single RPs. More longitudinal prospective studies are needed to enable a profound investigation of the association of RPs, behavioral problems, and potential confounders. Although we included a set of study-specific moderators in the meta-regressions, the inclusion of other essential moderators, such as maternal depression, preterm birth, parent-infant interaction, childhood trauma or childhood attachment was limited because comparable information of potentially relevant confounders was often lacking.

### Clinical implication

From a clinical perspective, our findings highlight the need for a better understanding of predictors of childhood behavioral problems and clinical disorders. The results suggest the importance of early monitoring, detection, and intervention for families with an infant affected by RPs to prevent the development of further behavioral problems. From a primary health care perspective, this is crucial information for pediatricians and parental counseling in childcare.

Overall, the knowledge about the impact of RPs on later behavioral problems should be used to develop and evaluate specific prevention programs focusing, for example, on parent-infant psychotherapy (87, 88). The mutual perspective of RPs offers several optional starting points for interventions for disrupted parent-infant interactions to reduce parental stress and foster further child development. Strengthening parentś self-efficacy to adapt to their infant´s needs and difficulties is just as important as identifying and treating emotional distress, particularly postpartal depression or anxiety which might keep parents from adequately understanding or supporting their infant (70).

## Conclusion

The present findings provide a comprehensive view of the development of behavioral problems in children with RPs. Results showed a robust positive association with small to medium effect size between RPs in infancy and childhood problem behavior and indicate the importance of further prospective longitudinal studies on the association between infant RPs and child development.

Though we found no significant difference regarding single RPs compared to multiple RPs, these findings should be replicated longitudinally and promote further investigations and interventions for infants with a single RP as well. With the help of prevention programs, RPs could be identified and treated at an early stage, reducing long-term consequences. Moreover, untreated behavioral problems and clinical disorders are associated with high health care costs and represent a relevant burden for affected families (89). Therefore, family counselors and pediatricians should assess potential crying, sleeping, and eating problems and the level of parental stress in a structured way at regular intervals during infancy to identify those who might be at risk of persistent RPs and developing behavioral problems in childhood.

## Data Availability

The original contributions presented in the study are included in the article/**Supplementary Material**, further inquiries can be directed to the corresponding author.
