# Supplementary material for: The impact of crying, sleeping, and eating problems in infants on childhood behavioral outcomes: A meta-analysis
Source: Front Child Adolesc Psychiatry. 2023 Feb 14;1:1099406. doi: 10.3389/frcha.2022.1099406 (PMC11732157; doi:10.3389/frcha.2022.1099406)
Supplement: Supplementary file 1 [file Datasheet1.pdf]

Online Supplementary Material

The impact of crying, sleeping and eating problems in infants on childhood behavioral outcomes: A meta-analysis

Britta Galling, MD[1,2*]; Hannah Brauer[2*]; Pia Struck[3]; Amanda Krogmann[4]; Mirja Gross-Hemmi, PhD[5],

Alexander Prehn-Kristensen, PhD[2,6*]; Susanne Mudra, MD[7*]

***** both authors contributed equally

Institutional affiliation:

[1] Department of Child and Adolescent Psychiatry, Psychosomatic Medicine and Psychotherapy, Charité-Universitätsmedizin Berlin, Berlin, Germany
[2] Department of Child and Adolescent Psychiatry and Psychotherapy, Centre for Integrative Psychiatry, School of Medicine, Kiel, Germany
[3] Department of Psychology, University of Hildesheim, Germany
[4] University Hospital Hamburg-Eppendorf, Hamburg, Germany
[5] Swiss Paraplegic Research, Guido A. Zäch Institute, Nottwil, Switzerland
[6] Department of Psychology, Faculty of Human Sciences, MSH Medical School Hamburg - University of Applied Sciences and Medical University, 20457 Hamburg,
[7] Department of Child and Adolescent Psychiatry, Psychotherapy and Psychosomatics, University Medical Center Hamburg-Eppendorf, Hamburg, Germany

Corresponding author: Britta Galling, Department of Child and Adolescent Psychiatry, Psychosomatic Medicine and Psychotherapy, Charité-Universitätsmedizin Berlin, Germany. E-Mail: britta.galling@gmail.com

Fig. S1 Forest Plot of standardized mean difference (SMD) for overall behavioral problems or ADHD in children with single regulatory problem in infancy vs. healthy controls.

Fig. S2 Forest Plot of standardized mean difference (SMD) for overall behavioral problems or ADHD in children with multiple regulatory problem in infancy vs. healthy controls.

Fig. S3 Forest Plot of standardized mean difference (SMD) for overall behavioral problems or ADHD in children with single RP vs. multiple regulatory problems.

Table S1 Detailed study and sample Characteristics

Table S2 Results of the study quality assessment using the Newcastle-Ottawa Quality Assessment Scale (NOS)

Table S3 Co-Primary Outcomes and specific behavioral problem outcome categories

Table S4 Subgroup analyses and Meta-regressions of secondary Outcomes

Table S5 Subgroup analyses and Meta-regressions of other Outcomes

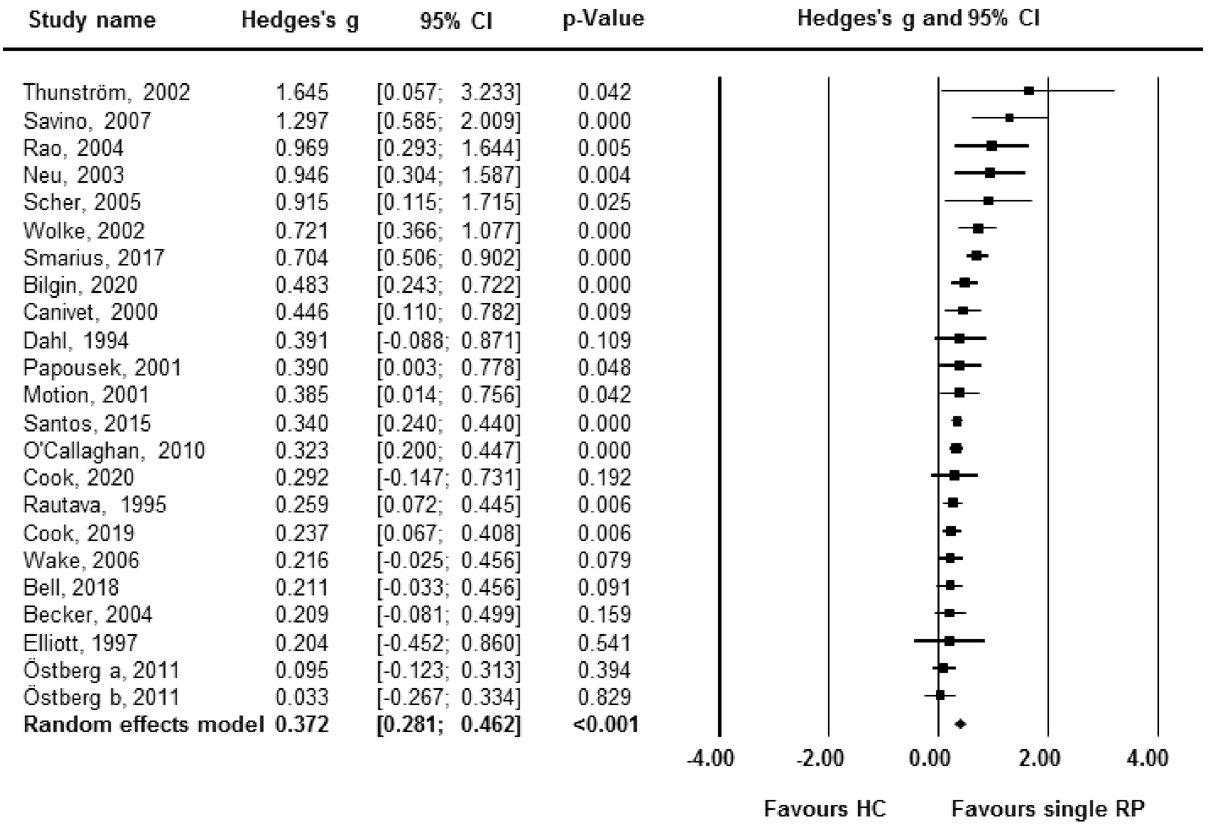

Fig. S1 Forest Plot of standardized mean difference (Hedge's g) for overall behavioral problems in children with single regulatory problem in infancy vs. healthy controls. Black whiskers indicate 95% confidence interval (CI), sizes of boxes are proportional to the study weight.

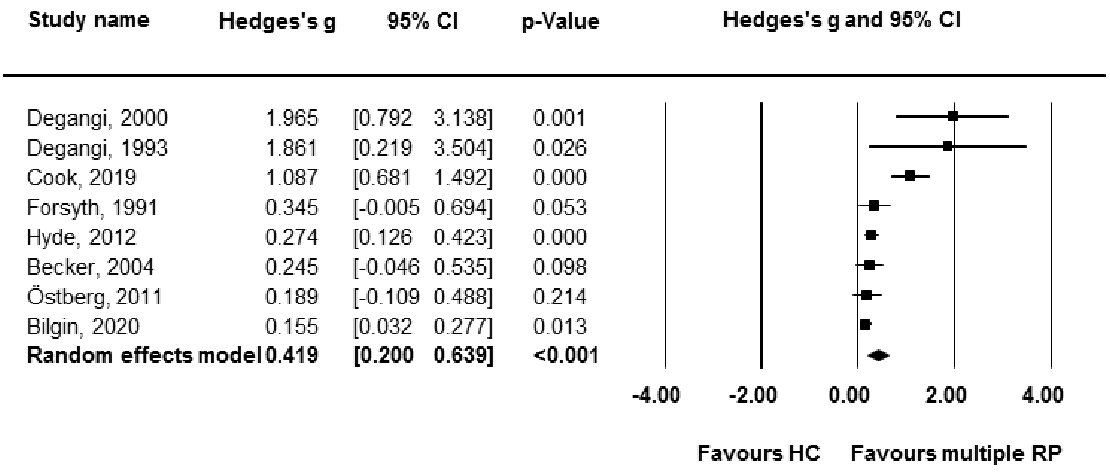

Fig. S2 Forest Plot of standardized mean difference (Hedge's g) for overall behavioral problems in children with multiple regulatory problem in infancy vs. healthy controls. Black whiskers indicate 95% confidence interval (CI), sizes of boxes are proportional to the study weight.

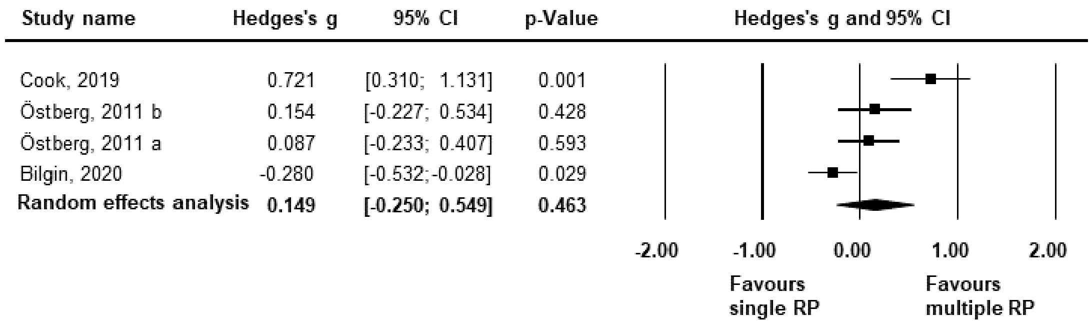

Fig. S3 Forest Plot of standardized mean difference (Hedge's g) for overall behavioral problems in children with single vs. multiple regulatory problems. Black whiskers indicate 95% confidence interval (CI), sizes of boxes are proportional to the study weight.

Table S1: Detailed Study and Sample Characteristics

Study	StQ (NOS)	N (BL) RP	N (BL) Control	Age (mean) BL (m)	Age (mean) f/u (years)	Sex Male (%)	Sample<br>- clinical sample vs. community<br>- inclusion criteria	BL assessment: Infant RPs	BL assessment: Parents	Analyzed follow-up outcomes<br>- Reported outcomes (instruments)<br>- Categorization of the outcomes (meta-analysis)	Other outcomes at follow-up
Crying											
**Bell 2018 (Australia)**	7	99	182	1.7	2	51.25	- clinical sample<br>- Wessel criteria	- D	- EPDS (PPD)	- CBCL<br>- EXT; INT	- PR; BISQ<br>- crying, sleeping, eating problems; temperament
**Canivet 2000 (USA)**	7	52	102	3	4	NR	- community sample (ft, ≥ 2500g)<br>- Wessel criteria	- PI/ D	NA	- RCBQ<br>- EXT; INT	- EAS; Q; CBCL<br>- temperament; eating behavior; sleep problems; psychosomatic complaints
**DeSantis 2004 (USA)**	4	165	-	2	5.6	NR	- clinical sample<br>- n hours crying / fussing	- PI/D	NA	- CBCL<br>- overall BP; EXT; INT; ADHD	- eating behavior; sleep problems; psychosomatic complaints
**Elliott 1997 (Canada)**	6	10	72	1.8	3	54.88	- community sample<br>- Wessel criteria	- Q	NA	- CBCL<br>- overall BP	- NCATS; FFFS<br>- MII; family functioning
**Neu 2003 (USA)**	5	20	20	2.5	7	50	- clinical sample<br>- crying for ≥2.8 h/d, ≥3 days	- PI/Q	NA	- CBCL; DICA-R<br>- EXT; INT; ADHD	-BSQ; MFFT; WISC-III; PSI<br>- temperament; cognitive style; intelligence; parenting stress, MII RP
**Papousek 2001 (Germany)**	6	83	57	4.1	2.5	NR	- clinical sample<br>- Wessel criteria	- D	- PIR-GAS (MII)<br>- EPDS (PPD)	- CBCL<br>- EXT; INT	-PR; ICQ<br>- crying, fussing and sleep problems; temperament
**Rao 2004 (Norway)**	7	63	264	9		NR	- community sample (ft infants)<br>- daily uncontrolled crying, ≥2 wks	- PI/Q	NA	- PIC<br>- ADHD	- WPPSI-R; PDMS<br>- intelligence; children's development
**Rautava 1995 (Finland)**	6	338	866	3	3	NR	- community sample<br>- questionnaire-based colic disorder	- Q/EO	NA	- PR<br>- EXT	- DDST; CBCL<br>children's development; behavior/ emotional problems family functioning
**Santos 2015 (Brazil)**	7	437	3237	3	4	52	- community sample<br>- >avg crying as same age	- mother report	- SRQ-20 (maternal MH)	- CBCL<br>- overall BP; EXT; INT	NA
**Savino 2005**	7	52	51	2	10	46.88	- clinical sample	- Q	NA	- clinical evaluation	PR

**(Italy)**							- crying avg 4h/d, >4 d/wk			- EXT	sleep disorder, fussiness-temperament
**Smarius 2017 (Netherlands)**	7	102	3287	3	5	50.25	- community sample (singleton birth, ft infants)<br>- crying avg ≥3h/ d/wk	- Q	- CES-D (PPD)<br>- SR (maternal distress)	- SDQ<br>- overall BP; EXT; INT	- PAS; SMFQ; DASS-21<br>children's generalized anxiety and mood; maternal stress
**Wolke 2002 (Germany)**	5	101	64	3.8	9.7	50	- clinical sample<br>- modified Wessel criteria	- D	NA	- SDQ<br>- overall BP; EXT; INT; ADHD	- PR<br>- eating behavior; sleep problem; temperament
Sleeping											
**Cook 2019 (Australia)**	7	446	647	12	5; 11	50.59	- community sample<br>- questionnaire-based sleep problem	- Q	- K6 (psych. distress)	- SDQ<br>- overall BP	NA
**Cook 2020 (Australia)**	9	283	360	7	4; 10	52.53	- community sample<br>- awakenings ≥3 times/night in the last wk	- PI/Q	- EPDS (PPD)	- DAWBA<br>- ADHD; EXT; INT	- SCAS-P; SDQ<br>- anxiety disorder; conduct problems, emotional symptoms, hyperactivity
**O'Callaghan 2010 (Australia)**	7	754	2943	6	5; 14	56.06	- community sample<br>- sleeplessness most days/ a few times a wk	- mother report		- CBCL<br>- ADHD	- PR<br>- sleep problem
**Price 2012 (Australia)**	4	225	-	7	6	54.67	- community sample (GA ≥32 wks)<br>-parent-reported sleep problem	- Q	- EPDS (PPD)	- SDQ<br>- overall BP; EXT; INT	- CSHQ; HRQoL<br>- Sleep Problem; psychosocial and physical quality of life
**Scher 2005 (Israel)**	6	13	12		3.5	NR	- community sample (ft, healthy infants)<br>- night waking and settling difficulties	- Q	NA	- CBCL<br>- overall BP	- PR<br>- sleep problem
**Thunström 2002 (Sweden)**	7	27	25	8.5	5.5	*16	- community sample (ft infants, GA ≥37 wks)<br>- ≥15min. to fall asleep; awakenings ≥3 times/ night ≥5 nights/wk. for ≥6 m	- PI/D	- PSI (psychosocial situation)	- PI; CI<br>EXT; ADHD	NA
**Zuckerman 1987 (UK)**	6	56		8	3	47	- community sample<br>- ≥1 h to settle after waking; awakenings ≥3 times/night; problem causing severe disruption to the mother's sleep	- PI	- maternal PPD (GHQ)	- BSQ<br>- EXT; INT; ADHD	- BSQ<br>- eating problem, sleep problems, fearful, peer-sibling relationship
**Östberg 2011 (Sweden)**	3	125	227	13	7.3	55.68	- clinical sample<br>- referred (sleep problem)	- NR	- PI (psycho-social situation)	- Connor's scale; RCBQ<br>- EXT; INT	- PR<br>- sleep and eating problem
**Wake 2006 (Australia)**	6	SP: 85<br>CP: 55	313	SP: 8<br>CP: 3	2		- community sample<br>- sleep and cry-fuss problems (PR)	- Q/D	NA	- CBCL<br>- overall BP; EXT; INT	- EPDS; PSI<br>- PPD; parental distress
Eating											

**Dahl 1992/ 1994 (Sweden)**	6	25	240; 38	7.8	4; 9.6	52.71	- clinical sample<br>- RTE and/or eating problem ≥ 1 m	- PI/EO	- EO; PI (psychosocial situation)<br>- MII	- Rutters PBQ; HSQ<br>- overall BP; ADHD	- GMDS; Q<br>- psychomotor development<br>- psychosocial situation; MII; eating behavior
**Motion 2001 (UK)**	5	28	10669	6	3.9	NR	- community sample<br>- eating difficulties for 4 wks.	- Q	NA	- SDQ<br>- ADHD; EXT	NA
**Östberg 2011 (Sweden)**	3	52	227	13	7.6	51.25	- clinical sample<br>- referred (eating problem)	- NR	- PI (psychosocial situation)	- Connor's scale; RCBQ<br>- EXT; INT	- PR<br>- seep and eating problem
Crying / Sleeping / Eating											
**Becker 2004 (Germany)**	9	MRP 55<br>SRP 120	264	3	2; 4.5; 8; 11	*27 (49)<br>*57 (47.5)	- community sample (obstetric and psychosocial risk sample)<br>- >1 SD ≥ mean for one factor=SRP<br>- >1 SD ≥ mean for irritable and somatic functioning=MRP	- PI/EO	- FAI (psychosocial situation)<br>- MII	- MPI<br>- overall BP; ADHD	NA
**Bilgin 2020 (Germany)**	7	MRP 388<br>SRP 81	977	5	8		- community sample (neonatal at-risk)<br>- cry duration ≥2 h/d, cry amount > avg, difficult to soothe<br>- wakes up ≥2 times/night, ≥15 min at night<br>- eating difficulties, vomiting, disordered mouth/tongue movement	- PI	NA	- CBCL<br>- ADHD	NA
**Cook 2019 (Australia)**	7	59	647	12	5; 11	49.79	- community sample<br>- presence and severity of sleep problems, excessive crying; coughed/ choked food<br>- global temperament, mood swing	- Q	- K6 (psychological distress)	- SDQ<br>- overall BP	NA
**DeGangi 1993 (USA)**	6	9	13	9.5	4	50	- clinical sample (ft infants)<br>- >20 min. to fall asleep, >2 waking/night [a]	- PI/ EO	NA	- SHQ<br>- overall BP; ADHD	- MSCA<br>- developmental outcomes
**DeGangi 1996 (USA)**	5	13	-	18.5	3	60	- clinical sample (ft, healthy infants)<br>- >20 min. to fall asleep, >2 waking/night [a]	- EO<br>- ITSC	NA	- CBCL; EO<br>- overall BP; ADHD	- MSCA; PSI<br>- developmental outcomes; temperament; sleep and eating problems; parental distress
**DeGangi 2000 (USA)**	5	22	38	18.5	3	58.33	- clinical sample<br>- >20 min. to fall asleep, >2 waking/night [a]	- PI<br>- ITSC	- PSI	- SHQ; CD<br>- overall BP	- CBCL<br>- total behavioral/emotional problems, EXT and INT
**Forsyth 1991 (USA)**	7	115	205	4	3.5	NR	- community sample<br>- parent-reported sleep problem	- PI	NA	- CBCL<br>- overall BP	- CVS; CCPS<br>- vulnerability; personality
**Hyde 2012 (Australia)**	7	480<br>483	4356<br>4263	6	5<br>14	52.30<br>51.77	- community sample	- Q	- DSSI (maternal anx & dep)	- CBCL<br>- overall BP; EXT; INT	NA

							- colic, sleeplessness, eating problems, overactivity		- MII		
**Östberg 2011 (Sweden)**	3	53	227	13	7.5	37.37	- clinical sample<br>- referred (sleep/eating problem)	NR	- PI (psychological dysfunction)	- Connor's sale ; RCBQ<br>- EXT ; INT	- PR<br>- sleep and eating problem

***** Of the RP group; a difficulties self-soothing/calming, hypersensitive to new stimulations, distress at eating time, refuses variety of food textures ; ADHD=Attention-deficit/-hyperactivity disorder; anx=anxiety; BISQ=Brief Infant Sleep Questionnaire; BL=baseline; BP=behavioral problem; BSQ=Behavioral Style Questionnaire; CBCL=Child Behavior Checklist; CCPS=Cohen Childhood Personality Scale; CD=clinical diagnosis; CI=Standardized Clinical Interview; COPE=Coping Inventory; CP=Crying problem; CSHQ=Child Sleep Habits CVS=Child Vulnerability Scale; D=diary; d=day DASS-21=Depression Anxiety Stress Scales; DAWBA=Development and Well-Being Assessment; DDST=Denver-Developmental-Screening-Test; dep=depression; DICA=Diagnostic Interview for Children and Adolescents; EAS=Emotionality, Activity, Sociability; EPDS=Edinburgh Postnatal Depression Scale; EO=Expert Observation; EXT=Externalizing Problems; FAI=Family Adversity Index; FFFS=Feetham Family Function Survey; ft=full term; f/u=follow-up; GA=gestational age; GI=gastrointestinal; GMDS=Griffiths Mental Developmental Scale; HRQoL=Health-related quality of life; HSQ=Home and School Questionnaire; ICQ=Infant Characteristics Questionnaire; INT=Internalizing problems; ITSC=Infant/Toddler Symptom Checklist; K6=Kessler 6 Psychological Distress Scale; m=months; min=minutes; MFFT=Matching Familiar Figures Test; MH=mental health; MII=Mother Infant Interaction; MPI=Mannheim Parent Interview; MRP=Multiple regulatory problems; MSCA=McCarthy scale of children's ability; N=number; NA=no assessment; NR=not reported; NCATS=Nursing Child Assessment Teaching Scales; NOS=Newcastle-Ottawa-Scale; PAS=Preschool anxiety scale; PBQ=Rutter Preschool Behavior Questionnaire; PDMS=Peabody developmental motor scale; PI=Standardized Parental Interview; PIC=Personality Inventory for Children; PIR-GAS=Parent-Infant Relationship Global-Assessment-Scale; PPD=postpartum depression; PPQ=Parent Psychomotor Questionnaire; PR=parent-report; PSI=Parenting Stress Index; Q=Questionnaire; RCBQ=The Rutters Children's Behaviour Questionnaire; RD=regulatory disorder; RP=regulatory problem; SCAS-P=Spence Children's Anxiety Scale for parents; SDQ=Strengths and Difficulties Questionnaire; SHQ=Sensorimotor History Questionnaire; SMFQ=Short Moods and Feelings Questionnaire; SP=Sleep Problem; SR=self-report; SRP=single regulatory problem; StQ= Study Quality; SQ=Sleep Questionnaire; SRQ-20=Self-Reporting Questionnaire; WISC=Wechsler Intelligence Scale for children.

Table S2 : Results of the study quality assessment using the Newcastle-Ottawa Quality Assessment Scale (NOS)										
**Study**		**Selection**				**Comparability**	**Outcome**			**NOS Score**
**Author**	**Design**	**Representativeness of the exposed cohort**	**Selection of the nonexposed cohort**	**Ascertainment of exposure**	**Demonstration that outcome of interest was not present at start of study**	**Comparability of cohorts**	**Assessment of outcome**	**Was follow-up long enough for outcomes to occur**	**Adequacy of follow up of cohorts**	
Becker 2004	PCS	Participants were not representative of infants with multiple RPs in the community. (Only infants born at risk were selected)	* Drawn from the same community as the exposed cohort	** Secure record (observation by trained raters); Structured interview	* Yes	** Matching control group (sex, only firstborn children with singleton birth, German-speaking parents, and no severe physical handicaps, obvious genetic defects or metabolic diseases) Study controls for gender, age, psychosocial risk factors and mother-infant interaction	* Record linkage (trained clinical psychologists and child psychiatrists)	* Yes, 10 years	* Complete follow up of all subjects	9
Bell 2018	PCS	*Infants were somewhat representive of the average of crying problems in the community (some infants were recruited from the emergency department)	Drawn from a different source (crying measure was different in the crying and the control group)	* Secure record (objective diary)	* Yes	** Matched for sex. Study controls for maternal postnatal depression, maternal education, Index of social disadvantage	Parent self-report	* Yes, 2.5 years	* Subjects lost to follow up unlikely to introduce bias - 13% lost to f/u	7
Bilgin 2020	PCS	Participants were not representative of infants in the community (only neonatal at-risk children)	* Drawn from the same community as the exposed cohort	* Structured interview	* Yes	*Study controls for gestational age and SES	*Record linkage (psychologists rated attention span) Parent self-report	* Yes, 8 years	* Subjects lost to follow up unlikely to introduce bias – 20% f/u	7
Canivet 2000	PCS	* Truly representative of the average of colic in infants in the community	* Drawn from the same community as the exposed cohort	* Secure record (objective diary or Structured interview)	* Yes	*Matched control group	Parent self-report	* Yes, 3 years	* Subjects lost to follow up unlikely to introduce bias - 8% lost to f/u	7

Cook 2019	PCS	* Somewhat representative of the average of multiple RPs in infants in the community	* Drawn from the same community as the exposed cohort	Mothers completed questionnaire	* Yes	** Study controls for maternal age, marital status, country of birth, Non-English-Speaking-Background, school completion, and low SES), child age, sex, birth weight, weeks' gestation, NICU or SCN admittance, and birth order, and maternal mental health (K6 score > 8)	Parent self-report	* Yes, 11 years	* Complete follow up of all subjects	7
Cook 2020	PCS	* Truly representative of the average of sleeping problems in infants in the community	* Drawn from the same community as the exposed cohort	* Structured interview	* Yes	** Study controls for maternal age at birth, maternal depression symptoms (EPDS score at 12 months), low SES, infant birth weight and gender	* Independent blind assessment	* Yes, 10 years	* Subjects lost to f/u unlikely to introduce bias - small number lost 22% at 4 years; 30% at 10 years	9
Dahl 1994	CRS; RCG	Participants were not representative of infants with eating problems in the community (only infants from the child health care units)	Drawn from a different source/ retrospective control group	** Secure record (medical records); Structured interview	* Yes	* Matched to children with respect to age, sex and residential area.	Parent self-report	* Yes, 9 years	* Subjects lost to f/u unlikely to introduce bias - 10% lost to f/u	6
Degangi 1993		Participants were not representative of infants in the community (only fussy babies were selected)	* Drawn from the same community as the exposed cohort	* Secure records	* Yes	Higher proportion of males in RP sample	* Record linkage	* Yes, 4 years	*Complete f/u of all subjects	6
Degangi 1996		Participants were not representative of infants in the community (only fussy babies were selected)	* Drawn from the same community as the exposed cohort	* Structured interview	67% of infants showed poor sustained attention at baseline	Higher proportion of females in RP sample	* Record linkage	* Yes, 2,5 years	*Complete f/u of all subjects	6
Degangi 2000		Participants were not representative of infants in the community (only fussy babies, troubles sleeping or eating, highly sensitive to sensory stimulation, or referred for clinic, were selected). Infants were	Drawn from a different source	* Structured interview	Infants showed attention problems at baseline	* Sample matched on age	* Record linkage	* Yes, 2,5 years	* Complete f/u of all subjects	5

		predominantly white, middle class, only 5% from minority populations								
Desantis 2004	CRS	Infants were not representative of colic in infants in the community. (Only infants from the Development Unit Colic Clinic were selected)	No control group	* Secure records	* Yes	No control group	Parent self-report	* Yes, 3,7 years	*Complete f/u of all subjects	4
Elliott 1997	PCS	* Truly representative of the average of crying problems in infants in the community	* Drawn from the same community as the exposed cohort	Self-report (questionnaire)	* Yes	*Sample matched on sex	Parent self-report	* Yes, 3,7 years	* Complete f/u of all subjects	6
Forsyth 1990	PCS	* Truly representative of the average of multiple RPs in infants in the community	* Drawn from the same community as the exposed cohort	* Structured interview	* Yes	* Study controls for sociodemographic data	Parent self-report	* Yes, 3,3 years	* Complete f/u of all subjects	7
Hyde 2012	PCS	* Truly representative of the average of multiple RP in infants in the community	* Drawn from the same community as the exposed cohort	Self-report (mother completed questionnaires)	* Yes	** Study controls for maternal education, maternal age, marital status at 6 months, recorded income, same partner at 6 months, mother-infant attachment, birth weight, breastfeeding duration, maternal anxiety and depression at 6 months	Parent self-report	* Yes, 3,3 years	*Subjects lost to f/u unlikely to introduce bias - small number lost (21%) at 5 years; 22% at 14 years	7
Motion 2001	PCS	* Truly representative of the average of eating problems in infants in the community	* Drawn from the same community as the exposed cohort	Self-report (questionnaire)	* Yes	*Study controls for preterm birth, birth weight <2500g, hospital clinic attendance, breast fed ≥ 1 month, use of pacifier	No description	* Yes, 3 years	No statement	5
Neu 2003	PCS	* Somewhat representative of the average of colic in the community	* Drawn from the same community as the exposed cohort	* Secure record (nurse record)	No, not reported	* Matched on sex, age	Parent self-report	* Yes, 6,5 years	Half of the original sample could not be located, and 8% refused to participate. No description of those lost	5

O'Callag han 2010	PCS	* Truly representative of the average of sleep problem in the community	* Drawn from the same community as the exposed cohort	* Structured interview	* Yes	** Study controls for birth weight, maternal age at baseline, maternal education at birth, maternal smoking at 6 months, breastfeeding, wanted pregnancy, feeling about caring for the baby, maternal depression at 6 months	Parent self-report	* Yes, 5,4 years	* Description provided of those lost	7
Östberg & Hagelin 2010	CRS; RCG	Infants were not representative of infants in the community. Selected group (referred to the hospital because of sleep and/or eating problems in infancy)	Drawn from a different source	No description	* Yes	Retrospective control group	Parent self-report	* Yes, 9 years	* Complete f/u of all subjects	3
Papouse k 2001	CRS; RCG	Infants were not representative of infants in the community. Selected group (the Munich Interdisciplinary Research and Intervention Program for Fussy Babies and crying problems	Drawn from a different source	* Structured interview	* Yes	** Matched to the clinical group by age, sex and birth status, biological, psychosocial risk factors, infant-maternal relationship: factors that affect the behavioral problem outcome	Parent self-report	* Yes, 1,3years	* Subjects lost to f/u unlikely to introduce bias - 17% lost to f/u	6
Price 2012	PCS	* Somewhat representative of the average of sleep problems in the community	No control group	Self-report (questionnaire)	* Yes	No control group	Parent self-report	* Yes, 4,7years	* Subjects lost to f/u unlikely to introduce bias -30% lost to f/u	4
Rao 2004	PCS	Infants were not representative of infants in the community (born small for gestational age)	Drawn from a different source	* Structured interview	* Yes	** Matched control group for durations of breast feeding, sociodemographic factors, and growth, study controls for race and SES, maternal characteristics	*Independent assessment	*Yes 5years	*Subjects lost to f/u unlikely to introduce bias-20% lost to f/u	7
Rautava 1995	PCS	Infants were not representative of infants in the community (only infants from nulliparous women)	* Drawn from the same community as the exposed cohort	*Secure record (nurse record) Self-report (questionnaire)	* Yes	* Matched to the clinical group by age, sex and birth status	Parent self-report	* Yes, 2,6 years	* 28% lost to f/u description provided of those lost	6

Santos 2015	PCS	* Truly representative of the average of colic in infants in the community (urban area of Pelotas, Southern Brazil)	* Drawn from the same community as the exposed cohort	Self-report (parent-reported/ one item question)	* Yes	** Study controls for family income, maternal age, maternal education, antenatal care, smoking in pregnancy, caffeine intake in third trimester, maternal mood symptoms in pregnancy	Parent self-report	* Yes, 3,6 years	* Subjects lost to f/u unlikely to introduce bias-10% lost to f/u	6
Savino 2005		* Somewhat representative of the average of colic in the community	Drawn from a different source	* Structured interview	* Yes	* Matched on age and gender	* Record linkage	* Yes, 9,7 years	* Subjects lost to f/u unlikely to introduce bias–6,8% lost to f/u	7
Scher 2005		* Truly representative of the average of sleep problem in infants in the community	* Drawn from the same community as the exposed cohort	Self-report (questionnaire)	* Yes	* Matched on age and gender	Parent self-report	* Yes, 2,5 years	* Subjects lost to f/u unlikely to introduce bias-19% lost to f/u	6
Smarius 2016	PCS	* Truly representative of the average of colic in infants in the community	* Drawn from the same community as the exposed cohort	Self-report (questionnaire)	* Yes	**Matched on age and gender; Study controls for maternal burden of infant care and maternal aggressive behavior	Parent self-report	* Yes, 4,5 years	* Subjects lost to f/u unlikely to introduce bias-25% lost to f/u	7
Thunström 2002	PCS	* Truly representative of the average of sleep problem in infants in the community	Drawn from a different source	* Secure record (pediatrician record; sleep diary)	* Yes	* Matched on age and gender	* Record linkage	* Yes, 4,5 years	* Subjects lost to f/u unlikely to introduce bias-25% lost to f/u	7
Wake 2006	PCS	* Truly representative of the average of crying and sleep problem in infants in the community	* Drawn from the same community as the exposed cohort	Self-report (questionnaire)	* Yes	* Matched on age and gender	Parent self-report	* Yes, 4 years	* Subjects lost to follow up unlikely to introduce bias-10% lost to f/u	6
Wolke 2002	RCG	Infants were not representative of infants in the community (only mothers who contacted the self-help group)	Drawn from a different source	* Secure record (diary)	* Yes	*Matched on age, gender, and sociodemographic factors; controlled for maternal depression	Parent self-report	* Yes, 4,5 years	* Subjects lost to f/u unlikely to introduce bias-21% lost to f/u	5

Zuckerman 1986	PCS	* Truly representative of the average of sleep problem in infants in the community	No control group	* Structured interview	* Yes	No control group	* Structured interview	* Yes, 2,4 years	* Unlikely to introduce bias - 10%subjects lost to f/u	6
The Newcastle-Ottawa Quality Assessment Scale (NOS) is a scale used for assessing the quality of nonrandomized studies in meta-analyses. In this assessment scale a study can be awarded with one star per category and a maximum of two stars for comparability. Asterisks indicate the number of stars a study achieved in this category. The overall result is indicated by the NOS score, where a score of ≥7 out of 9 indicates high study quality. CRS=clinical referred sample; EPD=Edinburgh Postnatal Depression; F/u=f/u; K6= Kessler 6 Psychological Distress Scale; PCS=prospective cohort study; RCG=retrospective control group; RP=regulatory problem; NICU=neonatal intensive care unit; SCN= special care nursery; SES=socioeconomic status.										

Table S3: Co-Primary Outcomes and specific behavioral problem outcome categories

	**CUMULATIVE INCIDENCE**							**COMPARISON OF ANY RP TO HEALTHY CONTROLS**						
**OUTCOME VARIABLES OR SUB-GROUP**	**N (n)**	**Incidence**	**Lower Limit**	**Upper Limit**	**Result: p-value**	**Heterogeneity p-value**	**I^2**	**N (n)**	**SMD**	**Lower Limit**	**Upper limit**	**Result: p-value**	**Heterogeneity p-value**	**I^2**
**OVERALL BEHAVIORAL PROBLEMS**														
**ALL STUDIES**	18 (2873)	0.233	0.179	0.298	n/a	<0.001	90.2	26 (31177)	**0.381**	**0.296**	**0.432**	**<0.001**	<0.001	56.9
**SAMPLE**														
Community sample	11 (2603)	0.201	0.153	0.261	n/a	<0.001	89.2	19 (30057)	**0.348**	**0.275**	**0.422**	**<0.001**	0.070	34.0
Clinical sample	7 (270)	0.300	0.184	0.450	n/a	<0.001	79.7	7 (1120)	**0.685**	**0.295**	**1.074**	**<0.001**	<0.001	78.2
**RPs DEFINITION**														
Strict[a]	16 (1682)	0.234	0.173	0.307	n/a	<0.001	86.8	24 (23806)	**0.381**	**0.269**	**0.491**	**<0.001**	<0.01	65.4
Lenient[b]	2 (1191)	0.233	0.150	0.344	n/a	<.001	93.5	2 (7371)	**0.333**	**0.256**	**0.411**	**<0.001**	0.84	0.00
**TYPE OF RPs**														
Crying problems	6 (609)	0.268	0.179	0.381	n/a	0.004	74.7	11 (9116)	**0.493**	**0.336**	**0.651**	**< 0.001**	<0.001	66.6
Sleeping problems	4 (1060)	0.231	0.137	0.362	n/a	<0.001	89.2	7 (5319)	**0.266**	**0.138**	**0.395**	**< 0.001**	0.206	29.1
Eating problems	0							3 (11228)	0.231	-0.021	0.482	0.072	0.256	26.6
Single RP[c]	13 (2182)	0.228	0.167	0.304	n/a	<0.001	90.6	23 (26789)	**0.377**	**0.283**	**0.470**	**<0.001**	<0.001	56.1
Multiple RP[c]	6 (687)	0.248	0.147	0.388	n/a	<0.001	83.5	8 (7474)	**0.415**	**0.197**	**0.634**	**<0.001**	<0.001	77.1
**AGE AT BASELINE**														
≤6 months	8 (892)	0.274	0.209	0.351	n/a	<0.001	75.6	13 (11012)	0.405	0.296	0.466	**<0.001**	<0.001	63.7
>6 months	10 (1981)	0.208	0.138	0.303	n/a	<0.001	92.1	13(21585)	0.355	0.223	0.487	**<0.001**	0.020	50.1
**META-REGRESSION**														
**Covariant**		**Coeff**							**Coeff**					
Mean age at baseline	17 (2843)	-0.017	-0.107	0.072	0.705			26 (31177)	-0.003	-0.041	0.036	0.891		
Mean age at follow-up	18 (2873)	-0.121	-0.242	0.001	0.051			26 (31177)	-0.018	-0.075	0.038	0.519		
% Male	16 (2698)	-0.080	-0.211	0.05	0.227			21 (18585)	-0.007	-0.066	0.051	0.367		
Sample size	**18 (2873)**	**-0.157**	**-0.291**	**-0.023**	**0.022**			26 (31177)	-0.004	-0.012	0.005	0.367		
NOS	18 (2873)	-0.058	-1.006	0.621	0.625			26 (31177)	0.001	-0.066	0.068	0.977		

Table S3: Co-Primary Outcomes and specific behavioral problem outcome categories

OUTCOME VARIABLES OR SUB-GROUP	N (n)	Incidence	Lower Limit	Upper Limit	Result: p-value	Heterogeneity p-value	I^2	N (n)	SMD	Lower Limit	Upper limit	Result: p-value	Heterogeneity p-value	I^2
	**CUMULATIVE INCIDENCE**							**COMPARISON OF ANY RP TO HEALTHY CONTROLS**						
**OTHER OUTCOMES**														
**EXTERNALIZING PROBLEMS**	**11**(1544)	**0.201**	**0.141**	**0.279**	**<0.001**	<0.001	88.6	16(25702)	**0.362**	**0.253**	**0.472**	**<0.001**	0.001	62.1
**INTERNALIZING PROBLEMS**	8(1443)	**0.160**	**0.120**	**0.209**	**<0.001**	0.001	72.8	**12** (13865)	**0.343**	**0.284**	**0.403**	**<0.001**	0.602	0.0
**ADHD**	**8**(1065)	**0.242**	**0.157**	**0.354**	**<0.001**	**<0.001**	83.6	11(15019)	**0.461**	**0.317**	**0.605**	**<0.001**	0.071	41.7

SMDs (standardized mean differences) > 0 indicate that a specific continuous outcome (e.g., symptom severity) was more pronounced in those with regulatory problems (RPs). Results indicating p-values ≤0.05 were considered statistically significant and are marked in bold; CI=confidence interval; Coeff=coefficient; N=number of comparisons; n=number of subjects; n/a=not applicable; NOS=Newcastle-Ottawa-Scale; SMD=standardized mean difference [a] Strict definition=structured interview or questionnaire based; [b]lenient definition=one-item parent report; [c]including separate single and multiple RPs within a study.

Table S4: Subgroup analyses and Meta-regressions of secondary Outcomes

COMPARISON OF SINGLE RP TO HEALTHY CONTROLS

**OUTCOME VARIABLES OR SUB-GROUP**	**N (n)**	**SMD**	**Lower Limit**	**Upper Limit**	**Result: p-value**	**Heterogeneity p-value**	**I²**
**ALL STUDIES**	**23** (26792)	**0.372**	**0.281**	**0.462**	**<0.001**	<0.001	57.5
Community sample	**17 (25859)**	**0.371**	**0.282**	**0.461**	**<0.001**	0.012	48.8
Clinical sample	**6 (933)**	**0.409**	**0.113**	**0.706**	**0.007**	0.001	74.5
**TYPE OF RP**							
Crying problems	**12 (9568)**	**0.462**	**0.318**	**0.605**	**<0.001**	0.001	65.9
Sleeping problems	**6 (4972)**	**0.273**	**0.126**	**0.419**	**<0.001**	0.120	42.7
Eating problems	3 (10949)	0.231	-0.021	0.482	0.072	0.255	26.9
**AGE AT BASELINE**							
≤6 months	**12 (10245)**	**0.445**	**0.315**	**0.576**	**<0.001**	0.002	62.7
>6 months	**11 (16547)**	**0.274**	**0.159**	**0.390**	**<0.001**	0.099	37.6
**META-REGRESSION**							
**Covariant**		**Coeff**					
Mean age at baseline	23 (26792)	**-0.024**	**-0.047**	**-0.002**	**0.036**		
Mean age at follow-up	23 (26792)	0.001	-0.32	0.035	0.949		
% Male	16 (13217)	-0.015	-0.053	0.022	0.412		
Sample size	23 (26792)	0.000	0.000	0.000	0.766		
NOS	23 (26792)	0.035	-0.025	0.096	0.253		

COMPARISON OF MULTIPLE RP TO HEALTHY CONTROLS

**OUTCOME VARIABLES OR SUB-GROUP**	**N (n)**	**SMD**	**Lower Limit**	**Upper limit**	**Result: p-value**	**Heterogeneity**	
						**p-value**	**I²**
**ALL STUDIES**	**8** (7474)	**0.419**	**0.200**	**0.639**	**<0.001**	<0.001	77.5
**SAMPLE**							
Community sample	**5 (7112)**	**0.365**	**0.147**	**0.583**	**0.001**	0.001	79.0
Clinical sample	3 (362)	1.209	-0.196	2.614	0.092	0.003	82.8
**AGE AT BASELINE**							
≤6 months	**3 (1910)**	**0.185**	**0.078**	**0.292**	**0.001**		
>6 months	**5 (5564)**	**0.742**	**0.266**	**1.219**	**0.002**		
**META-REGRESSION**							
**Covariant**		**Coeff**					
Mean age at baseline	**8 (7474)**	**0.059**	**0.010**	**0.109**	**0.019**		
Mean age at follow-up	8 **(7474)**	-0.008	-0.096	0.081	0.868		
% Male	6 (5883)	0.131	-0.035	0.297	0.122		
Sample size	8 (7474)	0.000	0.000	0.000	0.382		
NOS	8 (7474)	-0.013	-0.146	0.121	0.853		

COMPARISON OF SINGLE RP TO MULTIPLE RP

**OUTCOME VARIABLES OR SUB-GROUP**	**N (n)**	**SMD**	**Lower Limit**	**Upper Limit**	**Result: p-value**	**Heterogeneity**	
						**p-value**	**I²**
**ALL STUDIES**	**4 (961)**	**0.149**	**-0.250**	**0.549**	**0.463**	0.001	82.557
**SAMPLE**							
Community sample	**1 (433)**	**-0.280**	**-0.532**	**-0.028**	**0.029**	1.000	0.0
Clinical sample	3 (528)	0.305	-0.071	0.682	0.112	0.044	68.0
**AGE AT BASELINE**							
≤6 months	**1 (433)**	**-0.280**	**-0.532**	**-0.028**	**0.029**	1.000	0.0
>6 months	3 (528)	0.305	-0.071	0.682	0.112	0.044	68.0
**META-REGRESSION**							
**Covariant**		**Coeff**					
Mean age at baseline	4 (961)	0.073	-0.015	0.162	0.102		
Mean age at follow-up	**4 (961)**	**-1.178**	**-2.119**	**-0.236**	**0.014**		
% Male	-	-	-	-	-		
Sample size	4 (961)	-0.001	-0.005	0.002	0.420		
NOS	4 (961)	0.019	-0.023	0.269	0.885		

SMDs (standardized mean differences) > 0 indicate that a specific continuous outcome (e.g., symptom severity) was more pronounced in those with regulatory problems. Results indicating p-values ≤0.05 were considered statistically significant and are marked in bold; CI=confidence interval; Coeff=coefficient; N=number of comparisons; n=number of subjects; n/a=not applicable;

NOS=Newcastle-Ottawa-Scale; SMD=standardized mean difference [a] Strict definition=structured interview or questionnaire based; [b]lenient definition=one-item parent report; [c]including separate single and multiple RPs within a study.

Table S5: Subgroup analyses and Meta-regressions of other Outcomes

	CUMULATIVE INCIDENCE							COMPARISON OF ANY RP TO HEALTHY CONTROLS						
**OUTCOME VARIABLES OR SUB-GROUP**	**N (n)**	**Incidence**	**Lower Limit**	**Upper Limit**	**Result: p-value**	**Heterogeneity p-value**	**I^2**	**N (n)**	**SMD**	**Lower Limit**	**Upper limit**	**Result: p-value**	**Heterogeneity p-value**	**I^2**
**OTHER OUTCOMES – EXTERNALIZING PROBLEMS**														
**ALL STUDIES**	**11 (1544)**	**0.201**	**0.141**	**0.279**	n/a	<0.001	88.6	**16 (25702)**	**0.362**	**0.253**	**0.472**	**<0.001**	0.001	62.1
**SAMPLE**														
Community sample	**7 (1369)**	**0.205**	**0.132**	**0.303**	n/a	<0.001	91.6	**11 (24855)**	**0.303**	**0.224**	**0.383**	**<0.001**	0.207	24.9
Clinical sample	**4 (175)**	**0.190**	**0.086**	**0.370**	n/a	0.002	80.6	**5 (846)**	**0.667**	**0.234**	**1.100**	**0.003**	<0.001	84.3
**RP DEFINITION**														
Strict[a]	**10 (1107)**	**0.186**	**0.131**	**0.256**	n/a	<0.001	79.6	**15 (22027)**	**0.386**	**0.261**	**0.512**	**<0.001**	0.001	62.0
Lenient[b]	**1 (437)**	**0.375**	**0.331**	**0.422**	n/a	1.000	0.0	**1 (3674)**	**0.239**	**0.139**	**0.339**	**<0.001**	1.000	0.0
**TYPE OF RP**														
Crying problems	**5 (593)**	**0.243**	**0.143**	**0.382**	n/a	<0.001	80.8	**10 (9312)**	**0.358**	**0.233**	**0.483**	**<0.001**	0.010	58.3
Sleeping problems	**3 (306)**	**0.252**	**0.143**	**0.406**	n/a	0.014	76.7	1 (50)	1.125	-0.514	2.763	0.178	1.000	0.0
Eating problems	-	-	-	-	-	-	-	1 (10697)	0.243	-0.128	0.614	0.199	1.000	0.0
Single RP[c]	**9 (1152)**	**0.212**	**0.139**	**0.308**	n/a	<0.001	87.5	**13 (20348)**	**0.341**	**0.230**	**0.452**	**<0001**	0.028	47.6
Multiple RP[c]	**2 (519)**	**0.165**	**0.095**	**0.273**	n/a	0.169	47.2	**2 (4896)**	**0.722**	**0.077**	**1.367**	**0.028**	0.022	80.9
**AGE AT BASELINE**														
≤6 months	**5 (593)**	**0.243**	**0.243**	**0.382**	n/a	<0.001	80.8	**9 (8860)**	**0.394**	**0.258**	**0.531**	**<0.001**	0.014	58.2
>6 months	**6 (951)**	**0.176**	**0.115**	**0.260**	n/a	<0.001	82.8	**7 (16841)**	**0.325**	**0.116**	**0.533**	**0.002**	0.002	70.6
**META-REGRESSION**														
**Covariant**		**Coeff**							**Coeff**					
Mean age at baseline	**10 (1632)**	**-0.231**	**-0.315**	**-0.146**	**<0.001**			16 (25702)	-0.001	-0.029	0.028	0.960		
Mean age at follow-up	11 (1544)	0.046	-0.123	0.215	0.593			16 (25702)	0.033	-0.018	0.085	0.204		
% Male	**10 (1484)**	**-0.134**	**-0.236**	**-0.032**	**0.010**			12 (13454)	0.008	-0.055	0.071	0.808		
Sample size	11 (1544)	0.000	0.000	0.000	0.429			16 (25702)	0.000	0.000	0.000	0.532		
NOS	11 (1544)	-0.035	0.835	0.289	0.835			16 (25702)	0.012	-0.073	0.097	0.788		
**OTHER OUTCOMES – INTERNALIZING PROBLEMS**														
**ALL STUDIES**	8 (1443)	**0.160**	**0.120**	**0.209**	n/a	0.001	72.8	**12 (13865)**	**0.343**	**0.284**	**0.403**	**<0.001**	0.602	0.0
**SAMPLE**														
Community sample	**6 (1344)**	**0.167**	**0.121**	**0.227**	n/a	<0.001	79.7	**8 (13115)**	**0.346**	**0.281**	**0.410**	**<0.001**	0.850	0.0
Clinical sample	**2 (99)**	**0.133**	**0.079**	**0.216**	n/a	0.497	0.0	**4 (750)**	**0.414**	**0.178**	**0.649**	**0.001**	0.120	48.6
**RP DEFINITION**														

Strict[a]	**7 (1006)**	**0.167**	**0.120**	**0.228**	n/a	0.002	71.2	**11 (10191)**	**0.352**	**0.279**	**0.425**	**<0.001**	0.525	0.0
Lenient[b]	**1 (437)**	**0.128**	**0.100**	**0.163**	n/a	1.000	0.0	**1 (3674)**	**0.328**	**0.228**	**0.428**	**<0.001**	1.000	0.0
**TYPE OF RP**														
Crying problems	**3 (517)**	**0.217**	**0.092**	**0.432**	n/a	<0.001	88.0	**8 (8223)**	**0.342**	**0.271**	**0.414**	**<0.001**	0.795	0.0
Sleeping problems	**2 (281)**	**0.116**	**0.062**	**0.208**	n/a	0.165	48.2	-	-	-	-	-	-	-
Eating problems								-	-	-	-	-	-	-
Single RP[c]	**6 (924)**	**0.159**	**0.110**	**0.224**	n/a	**0.002**	73.8	**9 (8512)**	**0.345**	**0.274**	**0.416**	**<0.001**	0.850	0.0
Multiple RP[c]	**2 (519)**	**0.165**	**0.095**	**0.273**	n/a	0.169	47.2	**2 (4896)**	**0.499**	**0.142**	**0.856**	**0.006**	0.153	51.0
**AGE AT BASELINE**														
≤6 months	**3 (926)**	**0.217**	**0.092**	**0.432**	n/a	**<0.001**	88.0	**7 (517)**	**0.359**	**0.282**	**0.436**	**<0.001**	0.867	0.0
>6 months	**5 (1960)**	**0.150**	**0.114**	**0.194**	n/a	0.084	51.4	**5 (926)**	**0.325**	**0.195**	**0.455**	**<0.001**	0.176	36.8
**META-REGRESSION**														
**Covariant**		**Coeff**							**Coeff**					
Mean age at baseline	7 (1404)	-0.024	-0.148	0.099	0.700			12 (13865)	-0.005	-0.021	0.012	0.563		
Mean age at follow-up	8 (1443)	0.024	-0.124	0.173	0.747			12 (13865)	-0.009	-0.045	0.028	0.644		
% Male	7 (1383)	-0.041	-0.168	0.085	0.522			10 (13308)	0.009	-0.039	0.057	0.716		
Sample size	8 (1443)	0.000	0.000	0.000	0.936			12 (13865)	0.000	0.000	0.000	0.917		
NOS	8 (1443)	-0.042	-0.276	0.192	0.723			12 (13865)	0.029	-0.016	0.075	0.202		

OTHER OUTCOMES – ADHD

**ALL STUDIES**	**8** (1065)	**0.242**	**0.157**	**0.354**	n/a	**<0.001**	83.6	**11 (19055)**	**0.461**	**0.317**	**0.605**	**<0.001**	0.071	41.7
**SAMPLE**														
Community sample	4 (925)	0.175	0.129	0.234	n/a	0.154	42.9	8 (18655)	**0.417**	**0.271**	**0.562**	**<0.001**	0.139	36.4
Clinical sample	**4 (140)**	**0.321**	**0.177**	**0.509**	n/a	**0.019**	68.9	**3 (400)**	**0.630**	**0.199**	**1.061**	**0.004**	0.133	50.4
**RP DEFINITION**														
Strict[a]	**7 (311)**	**0.257**	**0.150**	**0.405**	n/a	**<0.001**	80.3	10 (15358)	**0.508**	**0.335**	**0.680**	**<0.001**	0.114	36.8
Lenient[b]	1 (754)	0.171	0.146	0.200	n/a	1.000	0.0	1 (3697)	**0.323**	**0.200**	**0.447**	**<0.001**	1.000	0.0
**TYPE OF RP**														
Crying problems	**3 (112)**	**0.243**	**0.083**	**0.531**	n/a	**0.005**	81.4	4 (3731)	**0.604**	0.398	0.809	<0.001	0.295	19.0
Sleeping problems	2 (779)	0.196	0.124	0.297	n/a	0.165	48.2	2 (3747)	**0.737**	**-0.464**	**1.938**	**0.229**	0.104	62.2
Eating problems	-	-	-	-	-	-	-	2 (10947)	0.353	0.060	0.646	0.018	0.104	62.2
Single RP[c]	**6 (1017)**	**0.210**	**0.125**	**0.332**	n/a	**<0.001**	85.7	9 (18714)	**0.459**	**0.317**	**0.601**	**<0.001**	0.151	33.4
Multiple RP[c]	**2 (48)**	**0.355**	**0.233**	**0.499**	n/a	**0.532**	0.0	2 (341)	**0.698**	**-0.350**	**1.746**	**0.192**	0.026	79.9
**AGE AT BASELINE**														
≤6 months	**3 (112)**	**0.243**	**0.083**	**0.531**	n/a	**0.005**	81.4	4 (3876)	0.490	0.277	0.704	**<0.001**	0.152	43.3
>6 months	**5 (953)**	**0.222**	**0.146**	**0.322**	n/a	**0.005**	72.9	7 (15179)	0.461	0.236	0.686	**<0.001**	**0.104**	43.0
**META-REGRESSION**														
**Covariant**		**Coeff**							**Coeff**					
Mean age at baseline	7 (1026)	-0.078	-0.263	0.107	0.409			11 (19055)	0.005	-0.052	0.061	0.875		
Mean age at follow-up	8 (1065)	0.028	-0.190	0.247	0.799			11 (19055)	-0.027	-0.085	0.032	0.376		

% Male	8 (1065)	-0.033	-0.178	0.133	0.657	10 (8358)	-0.025	-0.083	0.034	0.408
Sample size	8 (1065)	-0.001	-0.003	0.001	0.343	11 (19055)	0.000	0.000	0.000	0.394
NOS	8 (1065)	-0.232	-0.501	0.036	0.090	11 (19055)	-0.082	-0.181	0.017	0.104

SMDs (standardized mean differences) > 0 indicate that a specific continuous outcome (e.g., symptom severity) was more pronounced in those with regulatory problems. Results indicating p-values ≤0.05 were considered statistically significant and are marked in bold; CI=confidence interval; Coeff=coefficient; N=number of comparisons; n=number of subjects; n/a=not applicable; NOS=Newcastle-Ottawa-Scale; SMD=standardized mean difference [a] Strict definition=structured interview or questionnaire based; [b]lenient definition=one-item parent report; [c]including separate single and multiple RPs within a study.
